# Combining transcranial direct current stimulation with music therapy improves cognitive function in schizophrenia: study protocol for a randomized, double-blind, sham-controlled clinical trial

**DOI:** 10.3389/fpsyt.2025.1543789

**Published:** 2025-05-08

**Authors:** Yange Wei, Shanyuan He, Peng Luo, Hanshuo Su, Yuanle Chen, Shisen Qin, Zhongguo Zhang, Rongxun Liu, Dongshuai Wei, Juan Wang, Fei Wang, Chuansheng Wang

**Affiliations:** ^1^ Department of Early Intervention, Mental Health and Artificial Intelligence Research Center, The Second Affiliated Hospital of Xinxiang Medical University, Henan Mental Hospital, Henan Collaborative Innovation Center of Prevention and Treatment of Mental Disorder, Brain Institute, Henan Academy of Innovations in Medical Science, Xinxiang, China; ^2^ Peking University Sixth Hospital, Peking University Institute of Mental Health, National Health Commission (NHC) Key Laboratory of Mental Health (Peking University), National Clinical Research Center for Mental Disorders (Peking University Sixth Hospital), Beijing, China; ^3^ Department of Psychiatry, The Fourth People’s Hospital of Yancheng, Yancheng, China; ^4^ Department of Cardiovascular Medicine, The Seventh People’s Hospital of Zhengzhou, Zhengzhou, China; ^5^ Department of Early Intervention, Nanjing Brain Hospital, Nanjing Medical University, Nanjing, China; ^6^ Department of Psychiatry, Yale School of Medicine, New Haven, CT, United States

**Keywords:** schizophrenia, transcranial direct current stimulation, music therapy, cognitive functions, study protocol

## Abstract

**Background:**

Despite numerous pharmacological treatments, individuals with schizophrenia continue to exhibit significant residual cognitive impairments, adversely affecting the progression of the illness and their overall quality of life. Preliminary evidence indicates that transcranial direct current stimulation (tDCS) and music therapy (MT) may offer potential benefits for enhancing cognitive function in schizophrenia. This study aims to examine the synergistic efficacy of tDCS and MT on cognitive impairments in individuals with schizophrenia and to elucidate the potential mechanisms involved in this process.

**Methods:**

The study is designed as a randomized, double-blind, sham-controlled trial. All patients with schizophrenia will be randomly assigned to one of five groups: active tDCS combined with MT group, sham tDCS combined with MT group, active tDCS group, MT group, and a control group. The anodal electrode of tDCS will be positioned over the medial prefrontal cortex (mPFC), while the cathodal electrode will be placed over the visual cortex. MT will incorporate both Western Mozart and traditional Chinese classical music. The protocol involves 30-minute sessions conducted once daily, 5 days per week, for 4 consecutive weeks. The primary outcome measure is change in cognitive function, secondary outcomes include changes in psychotic symptoms, social function, and quality of life. Assessments will be evaluated at baseline (T0), after 2 weeks (T1), and after 4 weeks (T2). Furthermore, we will employ functional near-infrared spectroscopy (fNIRS) to examine hemodynamic changes on the cerebral cortex, and explore the neural effects of this combined treatment approach.

**Discussion:**

This study proposes an innovative non-pharmacological treatment protocol that combines tDCS targeting the mPFC with MT to improve cognitive impairments in schizophrenia. As a proof-of-concept study, it aims to provide empirical evidence for the effectiveness of this combined intervention. Moreover, this study seeks to elucidate the underlying neural mechanisms and offer a rigorous framework for future clinical trials, ultimately providing a novel therapeutic strategy for enhancing cognitive functions in patients with schizophrenia.

**Clinical trial registration:**

https://www.chictr.org.cn/, identifier, ChiCTR2400093161

**Trial registration details:**

The study is registered with https://www.chictr.org.cn/ under protocol registration number ChiCTR2400093161 (date of registration: 29. November. 2024). It was approved by the Research Ethics Committee of the Second Affiliated Hospital of Xinxiang Medical University (Approval Code: XYEFYLL-2024-82, Approval Date: 6 November 2024). Recruitment began in December 2024.

## Background

Schizophrenia is a severe and chronic psychiatric disorder with a lifetime prevalence of approximately 1% (1). It is characterized by positive symptoms, negative symptoms, and cognitive impairments such as deficits in working memory, verbal learning, attention, processing speed, and social cognition (2, 3). As a core symptom of schizophrenia, cognitive impairments typically manifest early in the disease course and may persist, significantly impacting patients’ social functioning and daily living abilities (4, 5). These cognitive impairments associated with schizophrenia (CIAS) encompass deficits in working memory, verbal learning, attention, processing speed, and social cognition (4, 5). Deficits in working memory and executive functions can hinder patients’ ability to plan, organize, and complete tasks, while impairments in social cognition may lead to difficulties in interpersonal relationships (6–8). Despite adequate pharmacological treatment, approximately 30% of individuals with schizophrenia persist in exhibiting cognitive impairments. Additionally, pharmacological treatments are often associated with substantial financial burdens for families, low compliance, and potential adverse effects, including extrapyramidal symptoms, endocrine, and metabolic disturbances (6). Consequently, the development of effective non-pharmacological alternatives for cognitive impairments is a major research priority.

Transcranial direct current stimulation (tDCS) is proposed as a noninvasive neuromodulatory strategy for schizophrenia due to its cost-effective, minimal adverse effects, and affordability (7). tDCS employs a constant, low-intensity direct current to modulate neuronal activity in the cerebral cortex (8). The potential mechanism involves changes in the resting membrane potential induced by hyperpolarization or depolarization depending on the polarity of the stimulation. Currently, tDCS predominantly targets the left dorsolateral prefrontal cortex (DLPFC) as the anodal site in schizophrenia research (9). The application of tDCS in individuals with schizophrenia typically involves positioning the anode on the left DLPFC, with the cathode placed either over the right supraorbital region or on the right DLPFC (10, 11). Notably, tDCS can modulate neural activity and enhance neuroplasticity, which can result in improvements in working memory, attention, and social cognitive abilities in patients with schizophrenia (12–15). A meta-analysis has demonstrated a significant positive impact of prefrontal tDCS on working memory, with a medium effect size (16).

Evidence from electroencephalogram, neuroimaging, and neuromodulation research has identified that the medial prefrontal cortex (mPFC) as a critical region implicated in cognitive dysfunction associated with schizophrenia (9, 17). Consequently, tDCS targeting the mPFC has the potential to enhance cognitive functions, including working memory, decision-making, and problem-solving (18). Although tDCS has shown promising results for the treatment of schizophrenia, recent studies indicate that its efficacy may be further enhanced when combined with other strategies (7).

Music therapy (MT) has been substantiated as a non-pharmacological treatment option for schizophrenia (19). MT is categorized into two forms: active MT, which encompasses activities such as singing, playing instruments, and music creation, and passive MT, which involves music listening (20). Its non-invasive nature, cost-effectiveness, and favorable tolerability make it a compelling adjunctive treatment for symptom management in schizophrenia. A systematic review including 13 randomized controlled trials with a total of 1,114 schizophrenia patients demonstrated improvements in cognitive functions, quality of life, social interest, and overall functioning after MT (19). MT has the potential to enhance cognitive functions and emotional states by facilitating the synchronization of activities across multiple brain regions and neural circuits (21). This effect has been observed in the mPFC, hippocampus, amygdala, insula, cingulate cortex, and hypothalamus (22). Consequently, MT may serve as a valuable adjunctive treatment for schizophrenia, particularly in alleviating cognitive deficits and promoting overall well-being through its synergistic effects (23).

Furthermore, the integration of tDCS with MT may yield greater cognitive improvements compared to the application of either treatment in isolation. tDCS has demonstrated efficacy in enhancing cognitive functions, especially in the mPFC, which is crucial for working memory, executive function, and self-referential cognition (17). The emotional and motivational enhancement effects associated with MT may enhance patient engagement and compliance with tDCS treatment, while tDCS may potentiate the effects of MT on brain functions (24). Based on this, we hypothesize that this integrated approach both targeting the mPFC may provide a novel therapeutic option for schizophrenia. Despite the growing body of literature on tDCS or MT, investigations into their combined application for the treatment of schizophrenia remain limited. Furthermore, clinicians and researchers have been explored objective assessment methodologies, particularly through neuroimaging techniques. Functional near-infrared spectroscopy (fNIRS) is an objective, portable, and cost-effective approach for evaluating cortical activity by monitoring blood oxygenation changes (25). Multichannel fNIRS instruments facilitate the measurement of alterations in specific brain regions and their temporal interrelationships (26). To date, no research has investigated the synergistic effects of tDCS and MT on cognitive impairments, nor the potential mechanisms underlying their combined application in schizophrenia.

This study protocol proposes a non-pharmacological approach by integrating tDCS with MT to investigate their synergistic effects on cognitive impairments in individuals with schizophrenia.This proof-of-concept study has three overall objectives. The first objective is to assess the efficacy of tDCS combined with MT on the treatment of cognitive impairments in patients with schizophrenia. We hypothesize that the integration of tDCS with MT will demonstrate greater effectiveness in ameliorating cognitive impairments compared to the application of either MT or tDCS. The second objective is to evaluate the efficacy of this integrated treatment approach on the positive and negative symptoms, as well as the quality of life, and social functioning. We expect that tDCS combined with MT will ameliorate both positive and negative symptoms and enhance overall functioning. The third objective is to investigate hemodynamic changes in the brain and explore the neural mechanisms involved in this combined treatment process by utilizing fNIRS during verbal fluency tasks (VFT). We propose the hypothesis that combined treatment will ameliorate prefrontal dysfunction, as measured by fNIRS, with more pronounced improvements observed in the combined group compared to those receiving tDCS or MT alone.

## Method

### Study design

**Figure 1:**
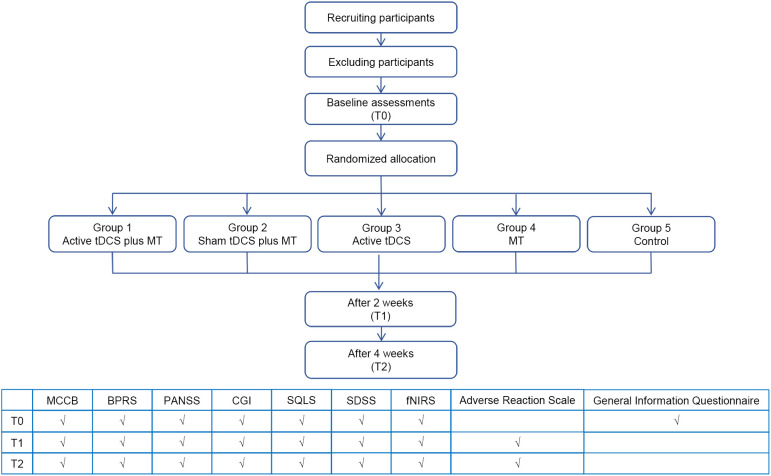
Flowchart of the study design. This flowchart outlines the various stages of the study as well as the corresponding group assignments. The table provides a detailed list of the assessments conducted at different time points. BPRS, Brief Psychiatric Rating Scale; CGI, Clinical Global Impressions; fNIRS, Functional Near-Infrared Spectroscopy; MCCB, MATRICS Consensus Cognitive Battery; MT, music therapy; PANSS, Positive and Negative Syndrome Scale; SDSS, Social Dysfunction Screening Scale; SQLS, Schizophrenia Quality of Life Scale.

This is a prospective, single-center, randomized, double-blind, sham-controlled study designed to assess the effects of tDCS combined with MT on the improvement of cognitive functions in patients with schizophrenia. Eligible participants will be recruited and randomly allocated into five groups in equal proportions (1:1:1:1:1): active tDCS combined with MT group (Group 1), sham tDCS combined with MT group (Group 2), active tDCS group (Group 3), MT group (Group 4), and control group (Group 5). Assessments will be conducted at baseline (T0), after 2 weeks (T1), and after 4 weeks (T2). The study design is depicted in **Figure 1**.

### Recruitment

Participants will be recruited from the Department of Psychiatry, the Second Affiliated Hospital of Xinxiang Medical University. The trial will be advertised via the hospital’s official website and various media platforms. Additionally, informational leaflets will be distributed within the Department of Psychiatry, and psychiatrists will provide an overview of the study’s content. Potential participants will receive both oral and written information regarding the study’s procedures as well as its potential benefits and risks. All participants and their legal guardians will sign the informed consent document. Detailed written informed consent is provided in **Supplementary Material**. In accordance with the Declaration of Helsinki’s Ethical Principles of Medical Research Involving Human Subjects, the study was approved by the Research Ethics Committee of the Second Affiliated Hospital of Xinxiang Medical University (Approval Number: XYEFYLL-2024-82, Approval Date: 6 November 2024) and registered with the China Clinical Trials Center (Registration Number: ChiCTR2400093161). The trial will be conducted at the Second Affiliated Hospital of Xinxiang Medical University. This study protocol is in accordance with the 2013 Standard Protocol Items: Recommendations for Interventional Trials (SPIRIT) statement guideline (27).

### Eligibility criteria

Participants will be assessed based on the inclusion and exclusion criteria detailed in **Table 1**. All patients routinely undergo the Drug Abuse Screening Test (DAST-10) and the Alcohol Use Disorders Identification Test (AUDIT) at admission, which are standard procedures at our hospital. During the enrollment, we will assess drug and alcohol abuse by reviewing the standardized test results and medical records of patients at admission. Severe drug and alcohol dependence, defined as a score of ≥3 on the DAST-10 (28) or ≥8 on the AUDIT (29) will be excluded from the study. The study will be discontinued under the following conditions: (1) the emergence of urgent medical issues; (2) the occurrence of serious adverse events and side effects; or (3) the participant or their family members express unwillingness to continue participation in the trial. Participants had been on a stable dose of antipsychotic medication for a minimum of two weeks, before the trial began. These medication doses remained unchanged throughout the study.

**Table 1:** Inclusion and exclusion criteria.

Inclusion criteria
1. Meets the clinical diagnostic criteria for schizophrenia according to DSM-5 for the current episode.
2. Patients who achieve a stable period through oral antipsychotic drug treatment are defined by the following criteria: a score of ≤5 on the items of delusion, hallucinatory behavior, exaggeration, and suspiciousness/victimization in the Positive and Negative Symptom Scale (PANSS), and a score of ≤4 on the PANSS conceptual disorganization.
3. Currently undergoing treatment with atypical antipsychotic medications, with equivalent doses of antipsychotic drugs calculated using the defined daily dose method.
4. Han ethnicity.
5. Ages between 18-55 years.
6. Normal hearing.
Exclusion criteria
1. Presence of brain organic lesions, intellectual disability, or other physical illnesses.
2. Frequent or persistent migraines.
3. Metal in skull and pacemaker.
4. Currently having epilepsy or a family history of epilepsy and other mental illnesses.
5. Severe drug and alcohol dependence.
6. Pregnant or lactating women.
7. Currently undergoing other neurostimulation therapy or evidence-based psychotherapy.

DSM-5: Diagnostic and Statistical Manual of Mental Disorders, 5th edition; PANSS, Positive and Negative Syndrome Scale.

### Randomization

The experiment will be conducted by an independent statistician, who was otherwise not involved in the study, utilizing random number tables to randomize the allocation of participants into one of five groups labeled ‘1, 2, 3, 4, 5.’ Specifically, these groups are coded as follows: active tDCS combined with MT (label = 1), sham tDCS combined with MT (label = 2), active tDCS alone (label = 3), MT alone (label = 4), and a control group (label = 5).

The randomization process was conducted using Microsoft Excel 2019. First, the 60 eligible patients were assigned unique identification numbers ranging from 1 to 60. Each number was then associated with a random value generated using the “=RAND ()” function in Excel. To ensure the random values remained fixed, the “Paste Special” function was used to convert the random values into static numbers. Subsequently, the list of patient numbers was sorted in descending order based on the generated random values. Following this sorting, the patients were sequentially allocated into five groups (Group 1 to Group 5), with 12 patients in each group, based on their order in the sorted list. This method ensured a completely random allocation of patients into the five study groups.

### Blinding

All participants and the psychiatrists conducting the assessments will remain blinded during the study. Regarding the implementation of blinding procedures, we have established the following considerations and measures. The specific group assignments of patients will be disclosed exclusively to the principal investigator. Furthermore, five distinct groups will be engaged to execute the treatment, with stringent restrictions in place to prevent inter-team communication concerning the details of their respective treatment. Each group will be informed solely of its own treatment plan and will remain unaware of both the specific group assignments and the plans of the other teams. During the experiment, the principal investigator will provide each group with the designated measures. Subsequently, each team will implement the experimental treatments on their respective patient cohorts. Patients remain blinded to the sequence allocation and are instructed to refrain from discussing group assignments, the treatments received, questionnaire completion, or any other elements of the experimental protocol among themselves. During the outcome measurement phase, personnel tasked with data collection and analysis are also blinded to the group assignments and specific measures. Next, the principal investigator will perform two unblinding procedures, one prior to and one following the data analysis. The initial unblinding will occur post data-lock, categorizing the data into Groups 1-5 without disclosing the actual alignment with Groups 1, 2, 3, 4, and 5, or their true group assignments. The second unblinding will be conducted after the completion of data analysis, elucidating the specific group identities corresponding to Groups 1, 2, 3, 4, and 5.

### Transcranial direct current stimulation

The tDCS will be administered using a battery-powered direct current stimulator (Model MBM-IV400, manufactured by Jiangxi Huaheng Jingxing Medical Technology Co., Ltd., located in Jiangxi, China) through two saline-soaked sponge electrodes (3.5 cm × 3.5 cm). According to the International 10–20 system, the anodal electrodes will be positioned over the mPFC, on the sites corresponding Fpz, whereas the cathodal electrode will be placed above the visual cortex, corresponding to Oz. The current intensity is set at 2 mA, and the electrode area is 4cm by 5 cm. The tDCS stimulation will occur once a day, 5 days per week, for 4 consecutive weeks. For the active tDCS condition, each session will involve delivering a 2 mA direct current for 30 minutes, with ramp-up and ramp-down periods of 30 seconds each. For sham tDCS, the stimulation will utilize the same active tDCS arrangement, with an intensity of 2 mA. However, the current will be applied only for the 30 s ramp-up phase at the beginning and 30 s ramp-down phase at the end of the stimulation.

### Music therapy

**Figure 2:**
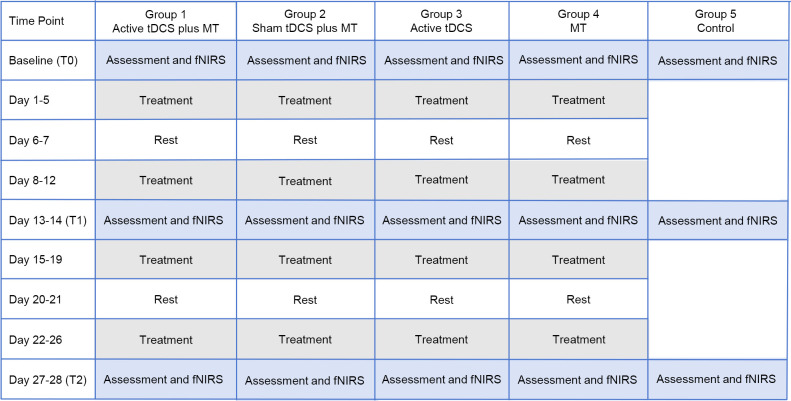
Study schedule for the participants in the randomized, double-blind, sham-controlled trial. fNIRS, Functional Near-Infrared Spectroscopy. MT, music therapy; tDCS, transcranial direct current stimulation.

For music selection, we will select western Mozart and Chinese classical music based on previous research (19, 30). For Western music, we will select Mozart’s Sonata K.448, a choice based on relevant literature that demonstrating its positive effects on improving symptoms in patients with schizophrenia (30). For traditional Chinese classical music, Pingsha Luoyan (Wild Geese Descending on the Sandbank), Chunjiang Huayueye (Moonlit River in Spring), Gaoshan Liushui (High Mountains and Flowing Water), and Shimian Maifu (Ambush from Ten Sides) will be selected according to a previous study by Lam et al. (19). For details, see **Table 2**. Participants in Group 1 (active tDCS combined with MT group), Group 2 (sham tDCS combined with MT group), and Group 4 (MT group) will engage in 20 sessions, each lasting 30 minutes, conducted once daily, five days per week over a four-week period. Notably, tDCS and MT will be conducted simultaneously in the Group 1 and Group 2. To minimize the potential confounding effects of temporal variables across the four groups (Groups 1, 2, 3, and 4), each treatment will be conducted within the same time period on the same day, with consistent start and end times. Participants in the control group (Group 5) will continue their regular medication treatment without receiving tDCS or MT. The detailed experimental design and operational procedures, please see to **Figure 2**.

**Table 2:** The standardize arrangement for music intervention.

Session	Music Category	Music Title
1	Western Mozart	Mozart’s sonata K.448
2	Chinese classical music	Pingsha Luoyan
3	Western Mozart	Mozart’s sonata K.448
4	Chinese classical music	Chunjiang Huayue Ye
5	Western Mozart	Mozart’s sonata K.448
6	Chinese classical music	Gaoshan Liushui
7	Western Mozart	Mozart’s sonata K.448
8	Chinese classical music	Simian Maifu
9	Western Mozart	Mozart’s sonata K.448
10	Chinese classical music	Erquan Yingyue
11	Western Mozart	Mozart’s sonata K.448
12	Chinese classical music	Zuiyu Changwan
13	Western Mozart	Mozart’s sonata K.448
14	Chinese classical music	Youlan
15	Western Mozart	Mozart’s sonata K.448
16	Chinese classical music	Saishang Qu
17	Western Mozart	Mozart’s sonata K.448
18	Chinese classical music	Xiyang Xiaogu
19	Western Mozart	Mozart’s sonata K.448
20	Chinese classical music	Meihua Sannong

### Outcomes

The study will utilize the Chinese version of the MATRICS Consensus Cognitive Battery (MCCB), which has demonstrated high efficacy in assessing cognitive function. To mitigate potential learning effects, various parallel versions of the MCCB will be employed at different assessment stages. Previous studies have demonstrated that the Chinese version of MCCB exhibits good test-retest reliability and validity, making it suitable for assessing cognitive dysfunction in patients with schizophrenia at each visit (31, 32). Cognitive function and psychotic symptoms will be assessed at baseline (T0), after 2 weeks (T1), and after 4 weeks (T2). According to the literature, intraclass correlation coefficient (ICC) will be used to assess the consistency of the assessors (33). The ICC values among assessors were 0.912, indicating the consistency was good. The procedure for the site visit is detailed in **Table 3**.

**Table 3:** Time schedule of screening, interventions and assessments.

	Recruitment	Baseline	Assessments	
			After 2 weeks	After 4 weeks
Time point		T0	T1	T2
Prescreening for eligibility, consenting and clinical interview				
Recruitment				
Eligibility screening	✓			
Informed consent	✓			
Allocation	✓			
Primary outcome assessment				
MCCB		✓	✓	✓
Second outcome assessment				
BPRS		✓	✓	✓
PANSS		✓	✓	✓
CGI		✓	✓	✓
SQLS		✓	✓	✓
SDSS		✓	✓	✓
fNIRS		✓	✓	✓
GIQ		✓		
**Safety**				
ARS			✓	✓

A checkmark (√) indicates the time point at which each assessment is carried out. ARS, Adverse Reaction Scale; BPRS, Brief Psychiatric Rating Scale; CGI, Clinical Global Impressions; fNIRS, Functional Near-Infrared Spectroscopy; GIQ, General Information Questionnaire; MCCB, MATRICS Consensus Cognitive Battery; PANSS, Positive and Negative Syndrome Scale; SDSS, Social Dysfunction Screening Scale; SQLS, Schizophrenia Quality of Life Scale.

The primary outcome measure is the change in the MCCB. It covers a total of seven different cognitive domains, including speed of processing, attention/vigilance, working memory, verbal learning, social cognition, reasoning and problem-solving (31). The MCCB is the gold standard measure of cognition in schizophrenia in clinical trials (34). It includes (1) Trail Making A Test, (2) Symbol Coding, (3) Hopkins Verbal Learning Test-Revised, (4) Digit Span Test, (5) Stroop Color-Word Test, (6) Spatial Span Test, (7) VFT, (8) Mazes, (9) Brief Visuospatial Memory Test-Revised, and (10) Continuous Performance Test-Identical Pairs.

The second outcome measures include the changes in general information questionnaire, Clinical Global Impressions (CGI) Scale, Brief Psychiatric Rating Scale (BPRS), Positive and Negative Syndrome Scale (PANSS), and fNIRS. Specifically, Clinical Global Impressions (CGI) is employed to evaluate both the severity of a patient’s condition and the efficacy of treatment. It comprises three components: the clinical global impressions-severity, which assesses the severity of illness; the clinical global impressions-improvement, which evaluates global impressions of improvement; and the efficacy index, which measures the overall efficacy of the treatment. BPRS is utilized to assess the severity of psychotic symptoms in patients, with higher scores indicative of more severe symptoms. PANSS includes a positive symptom scale and a negative symptom scale, each containing 7 items, as well as a general psychopathology scale with 16 items, culminating in a total of 30 items. Additionally, it incorporates 3 supplementary items designed to evaluate the risk of aggression. Each item on the PANSS has a definition and a specific 7-point operational rating scale. Both PANSS and BPRS will be used to assess psychotic symptoms from different perspectives (35, 36). By combining these two scales, we can more comprehensively capture changes in patients’ symptoms, thereby more accurately evaluating the effects of the treatment. The Social Dysfunction Screening Scale (SDSS) and Schizophrenia Quality of Life Scale (SQLS) is utilized to assess patient’s social function and quality of life, respectively.

A 48-channel fNIRS system (NirScan, Danyang Huichuang Medical Equipment Co. Ltd, China) will be employed in this study. The Chinese version of the VFT will be used to evaluate verbal fluency, working memory, verbal recall, attention, and retrieval (37). The VFT paradigm comprises a 30-second pre-task baseline period, a 60-second task period, and a 70-second post-task baseline period. During pre-task and post-task baseline periods, participants will be instructed to repeatedly count numbers from one to five. In the task period, subjects will be asked to verbally generate as many phrases as possible using three Chinese characters. A total of fifteen light source probes and sixteen light detector probes will be positioned on the bilateral frontotemporal cortex, ensuring a 3.0 cm distance between each light source and detector probe. In accordance with the 10/20 electrode placement system, the central probe will be aligned with FPz, while the lower boundary of the probe array will be extended from Fp1 to Fp2. Measurements of oxyhemoglobin, deoxyhemoglobin, and total hemoglobin will be conducted to assess hemodynamic changes. Raw fNIRS data will be preprocessed using the MATLAB toolkit Home 3. Five statistical measures of change in oxy-Hb signals, including mean, variance, skewness, kurtosis, and peak value will be calculated using the spatial average for all 48 channels. This yielded a total of 240 independent features for each subject.

### Sample size calculation

The sample size was calculated using G*Power software (G*Power, Version 3.1.9.7) based on a previous study (38). The study parameters include a Type I error rate of 0.05, a statistical power of 80%, and an effect size of 0.25. Based on these calculations, the required sample size was determined to be 45 participants using a repeated measures analysis of variance (ANOVA) model. Accounting for an estimated 20% dropout rate, a target sample size of 60 patients (12 per group) was established. Consistent with the previous literature by Emuk et al. (39), they also planned to include 12 participants per group, for a total of 60 participants. Here are the main reasons: When designing a pilot investigation where there is no prior information upon which to base the sample size, the recommendation would be a sample size of 12 per group as being appropriate. Three reasons for justifying a sample size of 12 per group will be given based on feasibility, gains in the precision about the mean and variance, and regulatory considerations (40). This sample size is expected to provide adequate statistical power to address the study objectives.

### Date management

Cognitive function and the severity of psychotic symptoms will be assessed by two clinical psychiatrists who remain blinded to the group allocation. All demographic data and scale-related information will be recorded in electronic CRFs and stored on a designated website. Access to these securely stored CRFs will be restricted to the project leader and the principal investigator. To ensure patient confidentiality, pseudonymization will be employed by substituting real patient names with unique identification numbers during data entry. Although the trial does not incorporate a data monitoring committee, oversight of the subject group is entrusted to a designated individual. Patient adherence to tDCS or MT sessions will be facilitated through notifications sent via WeChat.

### Statistical methods

The Shapiro-Wilk Test will be used to analyze whether the data conform to normality. If the quantitative data conform to a normal distribution, they will be expressed as the mean and standard deviation (SD); if they do not, they will be displayed as median values and interquartile range (IQR). Categorical data will be presented as counts with percentages. To evaluate differences in baseline characteristics between groups, t-tests, nonparametric tests, chi-square tests, Mann-Whitney U tests, and ANOVA will be employed appropriately. The Fisher exact test or χ² test will be used to compare adverse effects between groups.

In accordance with the CONSORT guidelines, analyses will be conducted following the intention-to-treat (ITT) principle, whereby all participants will be analyzed as originally randomized. A line graph will be utilized to depict values across different time points. To evaluate between-group and within-group differences over time for both primary and secondary outcomes, we will appropriately apply generalized linear mixed models and repeated measures ANOVA. A stepwise model selection process will be utilized to identify a parsimonious multivariable regression model. Age, gender and DDD doses of antipsychotic medications will be included as covariates in all models. The likelihood ratio test (LRT) will be utilized to assess the treatment effect by determining whether the coefficients for both the treatment and the interaction between time and treatment in the model are equal to zero. To account for multiple comparisons across multiple time points, p-values will be adjusted using the Bonferroni correction method. All statistical analyses will be conducted using SPSS 20.0. P < 0.05 is considered to be a statistically significant difference.

To minimize the impact of antipsychotic medications on clinical outcomes, several measures will be taken. First, all participants had been on a stable dose of antipsychotic medication for at least two weeks prior to the trial, and the doses of these medications remained unchanged throughout the study. This approach is consistent with previous studies (41), as maintaining a stable dose of antipsychotic medication helps reduce variability in drug effects during the intervention period. Second, we will adopt the Defined Daily Dose (DDD) method to standardize the doses of different antipsychotic medications across groups. By converting the actual doses of various antipsychotics into DDD units, we will compare and ensure that the drug doses are equivalent across different groups after randomization. Third, we plan to include the DDD doses as a covariate in the models to ensure the accuracy of the study and the reliability of the results.

### Data monitoring

The study was considered to involve no more than minimal risk to subjects, so the Institutional Review Board of the Second Affiliated Hospital of Xinxiang Medical University determined that a data monitoring committee was not required.

### Harms

To assess the safety and potential adverse effects of tDCS, this study will systematically analyze both serious and non-serious adverse events. Following each tDCS session, all participants will be required to complete the tDCS Adverse Events Questionnaire. Commonly reported side effects include sensations of tingling, mild erythema, itching, and discomfort at the site of stimulation. Participants will evaluate their adverse experiences on a scale ranging from 0 to 5. All adverse events will be recorded in the case report form (CRF). The tDCS device will be administered by a therapist with specialized training and experience in its application. Consequently, it is anticipated that participants will not encounter any significant health risks or adverse events. In the event that a serious adverse event does occur, the participant will be withdrawn from the trial, and the incident will be reported to the ethics committee.

### Auditing

The Principal Investigator will checked the CRFs and completed pro formas on a weekly basis. Research records in both paper and digital forms will be checked every two weeks for accuracy, and any discrepancies will be noted, discussed, and corrected.

### Ethics and dissemination

#### Research ethics approval

The study protocol, involving recruitment, consent and data collection tools was reviewed and approved by the Institutional Review Board of the Second Affiliated Hospital of Xinxiang Medical University Committee (Approval Code: XYEFYLL-2024-82). All individuals will sign an informed consent before they are enrolled in the study.

#### Protocol amendments

Any substantial modifications to the protocol will be submitted to the Research Ethics Committee of the Second Affiliated Hospital of Xinxiang Medical University. Upon approval, these protocol amendments will be formally communicated to the relevant parties and documented in the Chinese Clinical Trial Registry.

#### Confidentiality

All study staff are trained and certified in basic human subjects research protections by the Second Affiliated Hospital of Xinxiang Medical University Training Program to ensure confidentiality. Furthermore, the Principal Investigator provides continuous training and oversight to the evaluation coordinator to ensure the confidentiality and privacy of all participants and their data.

#### Data sharing

Raw data will be generated at the Second Affiliated Hospital of Xinxiang Medical University. Derived data supporting the findings of this study will be available from the corresponding author Chuansheng Wang upon request.

## Discussion

To the best of our knowledge, this represents the first randomized, double-blind, sham-controlled trial designed to assess the synergistic effects of tDCS and MT on cognitive impairments in patients with stable schizophrenia. Cognitive impairment constitutes a core symptom that substantially impacts the quality of life and social functioning of patients. In this context, we present an innovative study protocol to investigate the potential for cognitive improvement in schizophrenia through the combination of tDCS and MT. We hypothesize that the group receiving both active tDCS and MT will exhibit more pronounced effects compared to those receiving either tDCS or MT alone. If our hypothesis is validated, this study will offer an effective therapeutic strategy of cognitive impairments, thereby aiding clinical psychiatrists and potentially improving patients’ quality of life and social functioning. Considering the tDCS might be widely accessible for home use and the existing evidence supporting the efficacy of MT, this combination appears to be a promising approach for translational application in clinical practice. The implementation of this strategy could yield substantial clinical and economic benefits in the treatment of schizophrenia. This study will provide important references for future larger-scale clinical trials and is expected to promote the widespread application of combined tDCS and MT in clinical practice.

In this study, we intend to employ tDCS targeting the mPFC to ameliorate cognition deficits in schizophrenia. The mPFC plays a crucial role in the cognitive functions that are disrupted in schizophrenia, and its dysfunction is closely associated with cognitive impairments (42). Research by Pomarol-Clotet et al. identified the mPFC as a significant brain region of abnormality in schizophrenia using voxel-based morphometry, functional magnetic resonance imaging, and diffusion tensor imaging techniques (43). Correspondingly, enhancing mPFC activity through tDCS techniques has been suggested as a potential strategy for cognitive improvement in individuals with schizophrenia. Previous study found that active tDCS targeting the mPFC caused significant activation of the insula and decreased activation of the amygdala, which correlated with improvements in attention and working memory (16). Mechanistically, tDCS may facilitate these effects by inducing long-term potentiation (LTP)-like neuroplasticity changes (44). tDCS is posited to activate N-methyl-D-aspartic acid (NMDA) receptors in cortical neurons, and thereby modulating the release of neurotransmitters such as gamma-aminobutyric acid (GABA) and dopamine. This modulation potentially facilitates long-term potentiation (LTP) and long-term depression (LTD), enhances synaptic efficacy, and improves the efficiency of signal transmission within neural pathways (45–48). Furthermore, tDCS has been demonstrated to elevate the secretion of brain-derived neurotrophic factor (BDNF), a neurotrophin crucial for neuronal survival, neuroplasticity, and memory function (49). Collectively, tDCS targeting mPFC has emerged as a promising approach for cognitive enhancement in individuals with schizophrenia, potentially influencing the biological processes involved in LTP and neuroplasticity.

As an adjuvant treatment of schizophrenia, MT has demonstrated efficacy in enhancing cognitive function and psychological well-being, ameliorating psychotic symptoms, and improving quality of life (50, 51). From a clinical perspective, MT provides a safe and enjoyable therapeutic environment that facilitates increased patient engagement and treatment compliance. Research indicates that MT enhances arousal and emotional engagement, which can subsequently improve cognitive performance on memory-related tasks. Neuroimaging studies have demonstrated that MT activates brain regions associated with cognitive processing, potentially enhancing memory, attention, and executive functions (52). This effect has been observed in the mPFC, hippocampus, amygdala, insula, cingulate cortex, and hypothalamus (22). Notably, MT has been suggested to promote neuroplasticity in the brain, thereby facilitating the reorganization of sensory information storage and utilization (53). Mechanistically, the integration of somatosensory and auditory training can induce more pronounced plasticity changes in the auditory cortex compared to auditory training alone (54). This approach engages the brain’s emotional and cognitive regions, thereby enhancing neuroplasticity and strengthening neural connections. Functional neuroimaging studies provide evidence that MT can influence activity within limbic and paralimbic regions (55). Wang et al. demonstrated that a five-week MT alleviate overall symptoms, enhance social cognition in individuals with schizophrenia, and modulate neural oscillations, specifically decreasing theta oscillations in the parietal lobe and increasing gamma oscillations in the mPFC (56). Interestingly, mPFC mediates cognitive functions through its synaptic connections with these limbic and paralimbic brain regions (52). Collectively, these findings suggest that MT may enhance cognitive functions such as attention and working memory by modulating brain activity and neuroplasticity in the mPFC associated with cognitive processing.

The main innovation of this study is that the combination of tDCS targeting mPFC and MT may have synergistic effects to enhance cognitive improvement in patients with schizophrenia. tDCS targeting mPFC modulates t cortical excitability, thereby enhancing prefrontal activity and promoting neuroplasticity. MT can activate mPFC, which plays a crucial role in memory and attention processes, thereby potentially amplifying the effects of tDCS (57). The integration of tDCS and MT may present a novel therapeutic strategy designed to enhance cognitive rehabilitation efforts. Furthermore, this approach minimizes physical discomfort and potential side effects for patients and can be easily administered in a home setting, thereby increasing the convenience and accessibility of treatment. Additionally, the relatively low cost of tDCS and MT makes this treatment method more economically feasible compared to more complex neuromodulation techniques, such as transcranial magnetic stimulation. The integration of tDCS and MT is based on the observation that they share the similar mechanisms of action at the neural level, specifically through the modulation mPFC activity and neuroplasticity. The application of tDCS during music listening appears to enhance neural responses that are otherwise suppressed when tDCS is administered alone. Hence, if we can demonstrate combining tDCS with MT enhances cognitive improvement in schizophrenia, it might alleviate the disease burden and improve patient health outcomes.

As a non-pharmacological intervention, the combination of tDCS and MT can serve as a valuable adjunct to traditional antipsychotic medications. First, patients with schizophrenia often require long-term use of antipsychotic medications, which may lead to side effects such as extrapyramidal symptoms, endocrine disturbances, and metabolic issues. As non-pharmacological interventions, tDCS and MT may help mitigate these adverse effects, thereby improving patients’ overall quality of life. Second, the accessibility of tDCS and MT makes this combination a promising option for widespread clinical use. tDCS devices are relatively portable and easy to operate, and MT is simple to implement. If future research confirms the efficacy of this intervention, it could be widely adopted in various settings, including community health centers, rehabilitation facilities, and even home-based care. Third, the cost-effectiveness of tDCS and MT makes this combination a promising option for widespread clinical use. Future research should focus on identifying the optimal parameters for tDCS and MT, as well as developing standardized protocols for their use in clinical practice.

Regarding neuropsychological assessments, we will utilize fNIRS to monitor hemodynamic changes in the cerebral cortex to evaluate the synergistic effect. As a direct measure of neural activity, fNIRS can assess both local and network effects. Notably, fNIRS can be used to measure neural activity in specific brain regions, allowing for correlations with cognitive functions and the prediction of treatment response outcomes. The combined non-pharmacological treatment is expected to result in a more substantial alteration in prefrontal activity. We also expect that these changes will be associated with improvements in cognitive function. Consequently, the findings of this study will provide valuable insights into the potential mechanisms underlying combined treatment. Additionally, we will explore the mechanistic factors contributing to the significant differences observed among five groups. Blood and Zetore utilized positron emission computed tomography (PET) to detect alterations in cerebral activity through changes in regional cerebral blood flow (rCBF) during MT, they found that MT increased rCBF in the ventral striatum, orbitofrontal cortex, insula, and ACC, and decreased rCBF in the mPFC, hippocampus, and amygdala (58). Prior studies utilizing fNIRS have demonstrated that MT may assist individuals with memory impairments in improving episodic memory by reducing prefrontal activity (59). Taken together, this neurophysiological approach offers mechanistic evidence supporting the effectiveness of tDCS combined with MT, highlighting its potential as a non-pharmaceutical treatment for patients with schizophrenia in the long run.

Despite its advantages, this study has several limitations. Firstly, the relatively small sample size within each group may reduce the statistical power and limit the generalizability of the results. Secondly, this research will be conducted at a single site, which affects the external validity of the findings. Thirdly, the long-term effects and sustainability of the experimental treatment have not been comprehensively evaluated. Although the treatment period lasts for up to 4 weeks, the assessment of the long-term therapeutic effects after the treatment ends has not been conducted, which takes into account the limitation of the length of hospital stay. Therefore, the combined strategy warrants validation through larger, prospective multicenter studies to further investigate its long-term therapeutic effects on cognitive impairments in schizophrenia.

This randomized, double-blind, sham-controlled clinical study aims to demonstrate the synergistic effect of tDCS and MT on cognitive impairments, psychotic symptoms, and overall functioning in individuals with schizophrenia. Findings from this study may elucidate the potential mechanisms underlying the combined treatment on cognitive impairments and identify a novel target for schizophrenia. Given that the majority of schizophrenia patients experience a significant burden from pharmacological treatments, the implementation of non-pharmaceutical treatments holds considerable importance for both patients and clinical psychiatrists.

## Trial status

The preliminary research for the project began in December 2024.
