# Supplementary material for: Combining transcranial direct current stimulation with music therapy improves cognitive function in schizophrenia: study protocol for a randomized, double-blind, sham-controlled clinical trial
Source: Front Psychiatry. 2025 May 8;16:1543789. doi: 10.3389/fpsyt.2025.1543789 (PMC12100747; doi:10.3389/fpsyt.2025.1543789)
Supplement: Supplementary file 1 [file SupplementaryFile1.docx]

Supplementary Material

**Informed Consent Form**

**1. Introduce**

Schizophrenia is a severe and chronic psychiatric disorder with a lifetime prevalence of approximately 1%. Cognitive impairment constitutes a core symptom that substantially impacts the quality of life and social functioning of patients with schizophrenia. Despite adequate pharmacological treatment, approximately 30% of patients with schizophrenia persist in exhibiting cognitive impairments. Consequently, the development of effective non-pharmacological alternatives for cognitive impairments is a major research priority.

Preliminary evidence indicates that transcranial direct current stimulation (tDCS) and music therapy (MT) have the potential to enhance cognitive functions in schizophrenia. Considering the tDCS might be widely accessible for home use and the existing evidence supporting the efficacy of MT, this integrated approach appears promising for broad application in clinical practice. More importantly, the integration of tDCS and MT is based on the observation that they share the similar mechanisms of action at the neural level, specifically through the modulation mPFC activity and neuroplasticity. Therefore, we present an innovative clinical trial to investigate the potential enhancement of cognitive improvement in schizophrenia through the combination of tDCS and MT. Given the potential of these two non-pharmaceutical treatments, we would like to introduce this research to you.

**2. The name of the study and approval for its implementation**

The title of this study is " Combining transcranial direct current stimulation with music therapy improve cognitive function in schizophrenia: study protocol for a randomized, double-blind, sham-controlled clinical trial". The study was approved by the Research Ethics Committee of the Second Affiliated Hospital of Xinxiang Medical University (Approval Code: XYEFYLL-2024-82, Approval Date: 6 November 2024) and registered with the China Clinical Trials Center (Registration Number: ChiCTR2400093161). The study was conducted according to the Declaration of Helsinki.

**3. Objective**

This study has three overall objectives. The first objective is to assess the efficacy of tDCS combined with MT on the treatment of cognitive impairments in patients with schizophrenia. The second objective is to evaluate the efficacy of this integrated treatment approach on the positive and negative symptoms, as well as the quality of life, and social social functioning. The third objective is to investigate hemodynamic changes in the brain and explore the neural mechanisms involved in this combined treatment process by utilizing fNIRS during verbal fluency tasks (VFT).

**4. Transcranial direct current stimulation**

The tDCS is a non-invasive, effective, and cost-effective noninvasive neuromodulatory strategy for schizophrenia. tDCS can modulate neural activity and enhance neuroplasticity, which can result in improvements in working memory, attention, and social cognitive abilities in patients with schizophrenia.

**5. Music therapy**

Music therapy (MT) has been substantiated as a non-pharmacological treatment option for schizophrenia. It can serve as a valuable adjunctive treatment for schizophrenia, particularly in alleviating cognitive deficits and promoting overall well-being through its synergistic effects.

**6. Inclusion and exclusion criteria**

- 1. **Inclusion criteria**

1. Meets the clinical diagnostic criteria for schizophrenia according to DSM-5 for the current episode；
2. Patients have achieved stable schizophrenia through the administration of oral antipsychotic medications, as determined by the following evaluation criteria: scores for delusions, hallucinatory behaviors, exaggeration, and suspicion/victimization on the Positive and Negative Syndrome Scale (PANSS); a score of ≤5 on the general psychopathology scale for abnormal thought content; and a score of ≤4 on the PANSS for conceptual disorganization；
3. Currently undergoing treatment with atypical antipsychotic medications, with equivalent doses of antipsychotic drugs calculated using the defined daily dose method；
4. Han ethnicity；
5. Ages between 18-55 years；
6. A gender ratio of 1:1；
7. Normal hearing.
   1. **Exclusion criteria**
8. Presence of brain organic lesions, intellectual disability, or other physical illnesses；

2) Frequent or persistent migraines；

3) Metal in skull and pacemaker；

4) Currently having epilepsy or a family history of epilepsy and other mental illnesses；

5) Severe drug and alcohol dependence；

6) Pregnant or lactating women；

7) Currently undergoing other neurostimulation therapy or evidence-based psychotherapy.

**7. Intervention plan**

**7.1 Transcranial direct current stimulation plan**

The tDCS stimulation will occur 30-minute sessions, once a day, 5 days per week, for 4 consecutive weeks. The anodal electrodes will be positioned over the mPFC, whereas the cathodal electrode will be placed above the visual cortex. For the purpose of the active versus sham tDCS study, you will be randomly assigned to either the active tDCS group or the sham tDCS group.

**7.2 Music therapy plan**

MT will utilize western Mozart and Chinese classical music, including 30-minute sessions conducted once daily, 5 days per week, for 4 consecutive weeks. If you are assigned to either the active tDCS + MT group (Group 1) or the sham tDCS + MT group (Group 2), tDCS will be conducted concurrently with MT.

**7.3 Control plan**

If you are assigned to the control group (Group 5), you will continue with your daily medication without any treatment of tDCS or MT. The following outlines how the study will be administered:.

1. You will be screened to determine if you meet the inclusion criteria and do not meet the exclusion criteria.
2. Once confirmed to participate, you will be randomly assigned to one of the following groups: the active tDCS+ MT group (Group 1), sham tDCS + MT group (Group 2), active tDCS group (Group 3), MT group (Group 4), or the control group (Group 5). The assessors conducting the evaluations and yourself will not know which group you belong to until the study is completed. Before the first stimulation, we will collect your demographic data and conduct a baseline assessment.
3. The therapy will be carried out according to the plan of the assigned group.

During the study, you will need to report any adverse reactions.

1. You will be reassessed after the 10th and 20th intervention sessions.

Please note that the study will be discontinued if any of the following conditions occur:

1. Serious adverse events and side effects emerge, making it difficult to continue the study;

3) The patient or their family is unwilling to continue participating in the trial and withdraws the informed consent.

**7.4 Outcome assessment**

The primary outcome measure is the change in the MCCB, which comprises 10 tests covering seven different cognitive dimensions. It includes:

1. Trail Making A Test
2. Symbol Coding
3. Hopkins Verbal Learning Test-Revised
4. Digit Span Test
5. Stroop Color-Word Test
6. Spatial Span Test
7. VFT
8. Mazes
9. Brief Visuospatial Memory Test-Revised
10. Continuous Performance Test-Identical Pairs

The second outcome measures include:

1) General Information Questionnaire (GIQ)

2) Clinical Global Impressions Scale (CGI)

3) Brief Psychiatric Rating Scale (BPRS)

4) Positive and Negative Syndrome Scale (PANSS)

5) Social Dysfunction Screening Scale (SDSS)

6) Schizophrenia Quality of Life Scale (SQLS)

7) Adverse Reaction Scale (ARS)

We will conduct three fNIRS examinations at baseline (T0), after 2 weeks (T1), and after 4 weeks (T2). A 48-channel fNIRS system (NirScan, Danyang Huichuang Medical Equipment Co. Ltd, China) will be employed in this study. The Chinese version of the VFT will be used to evaluate verbal fluency, working memory, verbal recall, attention, and retrieval. During pre-task and post-task baseline periods, participants will be instructed to repeatedly count numbers from one to five. In the task period, subjects will be asked to verbally generate as many phrases as possible using three Chinese characters.

**8 What do you need to note?**

1) Provide accurate information about your medical history and current health status.

2) Inform the research physician of any discomfort you experience during this study.

3) Inform the research physician if you have participated in any other studies recently or are currently involved in other research.

4) Please adhere to the corresponding requirements of the study (such as not discussing with other participants what interventions you have undergone, etc.).

**9 The risk**

1) You may encounter the following risks: adverse reactions from the treatment, which may include pain, itching, mild transient skin redness, discomfort in the stimulated area, moderate fatigue, difficulty concentrating, and headaches.

2) You may experience psychological discomfort during the assessment process and distress

from being asked sensitive questions.

These are common risks in randomized clinical trials, but your information will be kept strictly confidential. If you are unwilling to bear the risks associated with being assigned to the control group (those who do not receive the intervention), you can refuse to participate. In the event of serious adverse effects, appropriate medical and nursing care will be provided until the symptoms subside, and you will not be charged any fees.

**10 Benefits**

1) You will not be required to pay any costs associated with this trial.

2) The study will provide you with feedback on your positive and negative symptoms, cognitive status, and other assessments and examinations, helping you gain a better understanding of yourself.

3) Participating in this study may accelerate your recovery from psychiatric symptoms and cognitive functions, among other conditions.

**11 Withdrawal of consent after participation**

Your participation in this study is entirely voluntary. You may refuse to participate, and declining to participate or withdrawing from the study at any time will not affect your current treatment, legal status, social services, or rights. There are two situations in which you might withdraw from the study early: one is if your doctor decides to terminate your participation for your benefit; the other is if you do not fully comply with the study protocol, in which case the researchers may also decide to discontinue your participation.

**12 If you have any questions about this research**

If you have any questions about this study, please contact our researchers:

Peng Luo Email: 50230101138@stu.xxmu.edu.cn

Shanyuan He Email: 50240101132@stu.xxmu.edu.cn

Hanshuo Su Email: 50240101121@stu.xxmu.edu.cn

However, we may not be able to respond to you or answer your questions when they involve the privacy of other participants or intellectual property rights.

**13 The system of this study**

Name of the medical institution: Department of Psychiatry, The Second Affiliated Hospital of Xinxiang Medical University.

Principal Investigator names: Yan Ge Wei, MD.

**Signing of Informed Consent Form**

 I have read this informed consent form.

 I have had the opportunity to ask questions and all my questions have been answered.

 I understand that participation in this study is voluntary.

 I can choose not to participate in this study, or withdraw at any time by notifying the researcher without discrimination or retaliation, and my medical treatment and rights will not be affected.

 If I need other treatments, or if I do not follow the research plan, or if there is any research-related injury or for any other reason, the research physician can terminate my continued participation in this study.

 I decide to consent to participate in this study.

Subject's Signature: Date: ______ year ______ month ______ day

Researcher's statement: I have accurately informed the subject of this document, he/she has read this informed consent form accurately, and I certify that the subject has had the opportunity to ask questions. I certify that he/she is voluntarily consenting.

Researcher's Signature: Date: ______ year ______ month ______ day
